# The causal relationship between the gut microbiota and acute pancreatitis: A 2-sample Mendelian randomization study

**DOI:** 10.1097/MD.0000000000038331

**Published:** 2024-05-31

**Authors:** Lin He, Haojun Luo, Yu Li, Yan Zhang, Li Peng, Yan Xu, Jing Lu, Jinzhi Li, Hang Liu

**Affiliations:** a Department of Pancreatitis Treatment Center, People’s Hospital of Deyang City, Deyang, China; b Department of Breast and Thyroid Surgery, The Second Affiliated Hospital of Chongqing Medical University, Chongqing, China.

**Keywords:** acute pancreatitis, causal effect, genetic, gut microbiota, Mendelian randomization

## Abstract

Several observational studies have reported a correlation between the gut microbiota (GM) and the risk of acute pancreatitis (AP). However, the causal relationship between them remains uncertain. We conducted a 2-sample Mendelian randomization (MR) study using pooled data from genome-wide association studies of 211 taxa (131 genera, 35 families, 20 orders, 16 classes, and 9 phyla) and AP patients. We evaluated the causal relationship between the GM and AP using methods such as inverse-variance weighting, MR-Egger, weighted medians, simple mode, and weighted mode. Cochran *Q* test, MR-Egger regression intercept analysis, and MR-PRESSO were used to examine the heterogeneity, multipotency, and outlier values of the variables, respectively. The reverse causal relationship between AP and the GM was assessed with reverse MR. In total, 5 gut microbial taxa were significantly associated with AP. The inverse-variance weighting results indicated that Acidaminococcaceae (odds ratio [OR]: 0.81, 95% confidence interval [CI]: 0.66–1.00, *P* = .045) and Ruminococcaceae UCG004 (OR: 0.85, 95% CI: 0.72–0.99, *P* = .040) were protective factors against the occurrence of AP. Coprococcus 3 (OR: 1.32, 95% CI: 1.03–1.70, *P* = .030), Eisenbergiella (OR: 1.13, 95% CI: 1.00–1.28, *P* = .043), and the *Eubacterium fissicatena* group (OR: 1.18, 95% CI: 1.05–1.33, *P* = .006) were risk factors for the development of AP. A comprehensive sensitivity analysis proved our results to be reliable. Reverse MR analysis did not indicate any causal relationship between AP and the GM. This study revealed a complex causal relationship between 5 GM taxa and AP, providing new insights into the diagnostic and therapeutic potential of the GM in AP patients.

## 1. Introduction

Acute pancreatitis (AP) is one of the most common digestive tract diseases worldwide.^[1]^ Pancreatic enzyme dysfunction resulting from AP may be caused by a variety of etiologies, with autolysis of the pancreatic alveolar cells followed by local pancreatic inflammation and extension of the inflammatory response to involve other organs or systems of the body. Globally, 34 cases of AP occur per 100,000 individuals annually, with an increasing incidence.^[2]^ As described in Atlanta (2012), AP can be divided into 3 grades: mild AP, moderately severe AP, and severe AP.^[3]^ While most cases are mild, approximately 20% of cases will progress to moderate to severe disease, resulting in a 30% mortality rate.^[4]^ There are several causes and obscure pathogeneses of AP, which have been extensively studied but still are not understood. Therefore, elucidating the pathogenesis of AP can aid in the development of better treatment strategies.

In the gut, thousands of organisms interact with one another to form complex gut microorganisms that, as the host evolves, play crucial roles in metabolism, immunity, development, and behavior.^[5]^ Increasing evidence in recent years suggests that the gut microbiota is strongly associated with the development of AP. Dysregulated secretion of pancreatic proteases during AP is caused by destruction of the pancreatic structure, leading to changes in gut homeostasis and the microbiota.^[6]^ Increased levels of Bacteroides and Proteobacteria and decreased levels of Firmicutes and Actinomyces were detected in AP patients compared with controls in a case-control study.^[7]^ Furthermore, the oral administration of *Lactobacillus brevis*-derived polyphosphate before the onset of AP in a mouse model attenuated the inflammatory response.^[8]^ However, the evidence presented has several limitations, including interspecies differences, small numbers of studies, and intrinsic flaws in observational studies, which make the true causal relationship between the gut microbiota and AP unclear; thus, further investigation is needed.

We used Mendelian randomization (MR) to assess the causal relationship between exposure and disease outcomes, using genetic variation to construct an exposure instrumental variable (IV).^[9]^ The distribution of genotypes from parents to children is random, so common confounding variables have no influence on the association between genetic variation and outcome, and a causal sequence is plausible.^[10]^ In this work, a double-sample MR study was conducted to evaluate the causal relationship between GM and AP using pooled statistical data from the genome-wide association study (GWAS) of the MiBioGen and FinnGen initiatives.

## 2. Material and methods

### 2.1. Two-sample MR design

**Figure 1.**
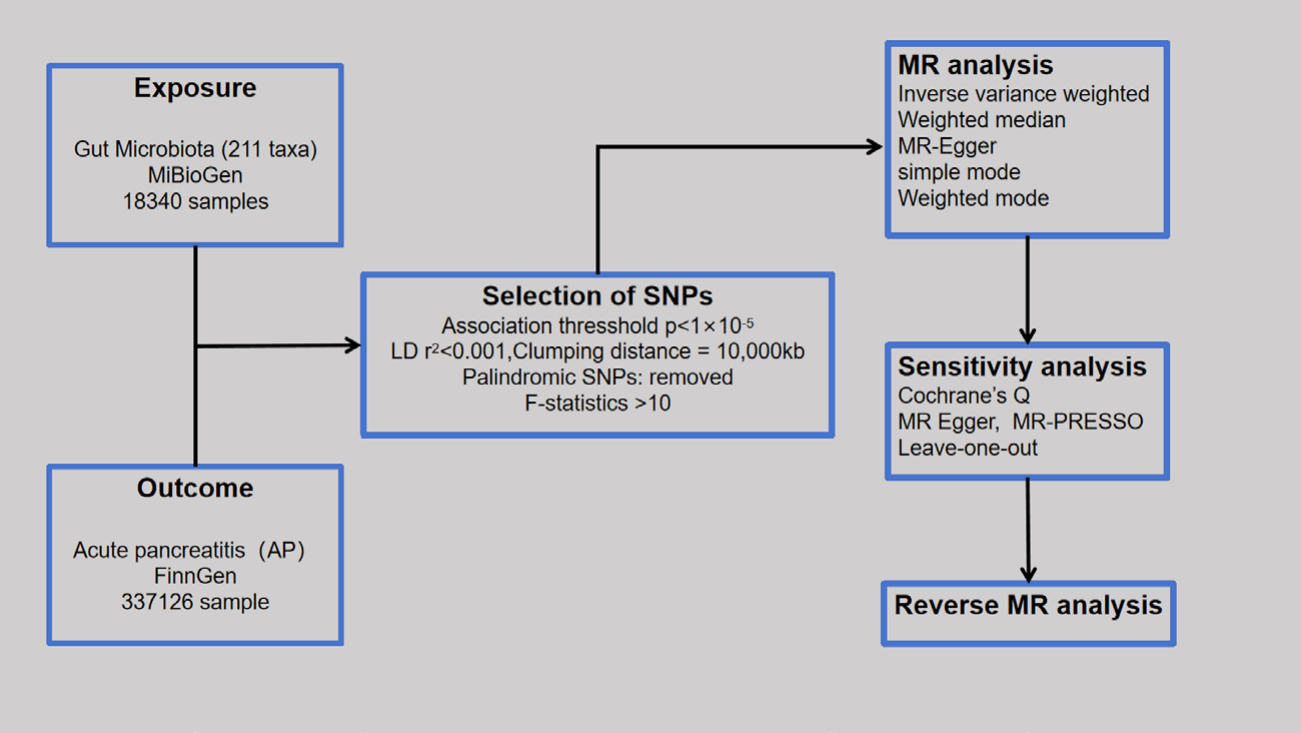
Overview of the Mendelian randomization framework used to investigate the causal effect of the gut microbiota on acute pancreatitis. MR = Mendelian randomization, SNP = single-nucleotide polymorphism.

This study investigated the causal relationship between 211 gut microbial taxa and AP using a double-sample MR assay. Statistical data on the gut microbiota and AP were summarized, and IVs were selected from substantial GWASs. To mitigate potential bias in outcomes, it was important to adhere to the following 3 principal assumptions of MR methods^[11]^: IVs must be significantly associated with exposure. IVs should affect outcomes only through exposure. IVs should not be associated with outcomes due to confounding factors. A summary of the study design is shown in Figure 1.

### 2.2. Data sources

The GWAS data for the gut microbiota were derived from the MiBioGen study,^[12]^ which is the largest, multiethnic, genome-wide meta-analysis of the gut microbiota to date. This study analyzed 16S rRNA gene sequencing profiles and genotyping data from 24 cohorts of 18,340 participants of European ethnicity from 11 countries, adjusted for age, sex, technical covariates, and genetic principal components for association analysis, ultimately determining data from 211 gut microbiota clusters at 5 classification levels (from the genus to the entry level). GWAS data for AP were obtained from the FinnGen study,^[13]^ which included 6223 cases and 330,903 controls from the Finnish population.^[11]^ In the FinnGen study, the International Classification of Diseases-10 (ICD-10) diagnosis code K85 was used to define AP. The GWAS data were obtained at https://r9.finngen.fi/. The populations involved in this MR study were all from European populations, thereby reducing the bias caused by population stratification.^[14]^

### 2.3. Selection of instrument variables

We systematically classified and analyzed 5 bacterial taxa and used the following criteria to ensure the accuracy and validity of causal relationships between GM and AP. SNPs that were significantly associated with GM were selected as IVs, and cutoff values of 1 × 10^−5^ were set as the standard.^[15]^ To ensure the independence of the selected IVs and to minimize the effects of linkage disequilibrium (LD), we performed aggregation analyses and set the aggregation parameters to *r*^2^ < 0.001 and an aggregation distance = 10,000 kb. LDs were estimated based on the European 1000 Genome Projects reference panel. 3. The allele sequence was deleted in A/T or G/C. A minor allele (MAF) of <0.01 SNP was removed. PhenoScanner V2 (http://www.phenoscanner.medschl.cam.ac.uk/) ruled out SNPs associated with potential confounders to ensure the second core hypothesis of MR. The deviation of the tool variable was reduced so that the *F*-statistic for each selected SNP was >10.^[16]^ The formula *F* = *R*^2^(n − *k* − 1)/*K*(1 − *R*^2^) was used, where n, *K*, and *R*^2^ represent the variance of sample size, number of IVs, and the exposure variance explained by the selected SNPs, respectively.

### 2.4. Statistical analysis

We evaluated the potential causal relationship between the gut microbiota and AP using a variety of effective methods, including inverse-variance weighting (IVW), weighted median, MR-Egger, simple mode, and weighted mode. We chose the IVW method, which was evaluated by combining Wald estimates for each SNP with weighted medians, simple modes, and weighted modes as auxiliary methods, as the main method for MR analysis.^[17–19]^ To further increase the robustness of the results, we performed a sensitivity analysis. First, we used Cochran *Q* test to test the heterogeneity of the selected SNPs. If heterogeneity existed (*P* < .05), we used the random-effects IVW method, whereas we used the fixed-effects IVW method.^[20]^ Second, we used MR-Egger regression to test for potential horizontal pleiotropy, with *P* values of <0.05 for intercepts where there might be horizontal pleiotropy for SNPs.^[21]^ The MR-PRESSO test was performed because of the low accuracy of the MR-Egger test in a few cases. In addition, to determine whether AP has a causal effect on the identification of significant bacterial species, we performed reverse MR analysis. All MR analyses were performed using R version 4.2.2, with the following software packages: “Mendelian Randomization,” “TwoSampleMR,” and “MR-presso.”

## 3. Results

### 3.1. Selection of IVs

SNPs significantly associated with 211 gut microbial taxa were screened for IVs using whole-genome locus significance analysis (*P* < 1 × 10^−5^). A total of 2249 SNPs were identified by removing LD and palindrome sequences. These clusters included 9 phyla (103 SNPs), 16 classes (180 SNPs), 20 orders (218 SNPs), 35 families (384 SNPs), and 131 genera (1364 SNPs). All IVs had *F* statistics >10, indicating a lower likelihood of weak instrument bias. This is detailed in Supplementary Table 1, Supplemental Digital Content, http://links.lww.com/MD/M668.

### 3.2. Two-sample MR analysis

In total, 5 gut microbial taxa were significantly associated with AP. We chose IVW as the primary study method, and preliminary MR analysis identified 5 gut bacterial populations as the study subjects. The relative abundance of 2 genetically predicted genera, Acidaminococcaceae (OR: 0.81, 95% CI: 0.66–1.00, *P* = .045) and Ruminococcaceae UCG004 (OR: 0.85, 95% CI: 0.72–0.99, *P* = .040), was inversely associated with risk. The relative abundances of the other 3 genera, Coprococcus 3 (OR: 1.32, 95% CI: 1.03–1.70, *P* = .030), Eisenbergiella (OR: 1.13, 95% CI: 1.00–1.28, *P* = .043), and the *Eubacterium fissicatena* group (OR: 1.18, 95% CI: 1.05–1.33, *P* = .006), were positively associated with the risk of AP. The results of other MR analyses (WM and MR-Egger) were consistent with the corresponding IVW analyses of the 5 categories (Table 1).

**Table 1:** MR results of causal links between gut microbiota and acute pancreatitis (*P* < 1 × 10^−5^).

Bacterial taxa	Nsnp	Method	*β*	SE	*P* value	OR (CI)	*F*-statistic
Acidaminococcaceae	7	IVW	−0.21	0.10	.045	0.81 (0.66–1.00)	21.29
Acidaminococcaceae	7	MR-Egger	0.01	0.33	.979	1.01 (0.53–1.92)	
Acidaminococcaceae	7	Weighted median	−0.18	0.14	.205	0.84 (0.64–1.10)	
Acidaminococcaceae	7	Simple mode	−0.15	0.21	.507	0.87 (0.58–1.29)	
Acidaminococcaceae	7	Weighted mode	−0.15	0.18	.432	0.86 (0.61–1.22)	
Coprococcus 3	8	IVW	0.28	0.13	.030	1.32 (1.03–1.70)	21.47
Coprococcus 3	8	MR-Egger	0.35	0.71	.636	1.43 (0.35–5.75)	
Coprococcus 3	8	Weighted median	0.29	0.17	.088	1.33 (0.96–1.85)	
Coprococcus 3	8	Simple mode	0.29	0.25	.289	1.34 (0.81–2.20)	
Coprococcus 3	8	Weighted mode	0.28	0.25	.289	1.33 (0.82–2.16)	
Eisenbergiella	11	IVW	0.13	0.06	.043	1.13 (1.00–1.28)	21.08
Eisenbergiella	11	MR-Egger	0.02	0.46	.967	1.02 (0.41–2.51)	
Eisenbergiella	11	Weighted median	0.12	0.08	.142	1.13 (0.96–1.32)	
Eisenbergiella	11	Simple mode	0.05	0.14	.705	1.06 (0.80–1.39)	
Eisenbergiella	11	Weighted mode	0.07	0.13	.584	1.08 (0.83–1.40)	
*Eubacterium fissicatena* group	9	IVW	0.17	0.06	.006	1.18 (1.05–1.33)	21.16
*E. fissicatena* group	9	MR-Egger	−0.01	0.31	.985	0.99 (0.54–1.83)	
*E. fissicatena* group	9	Weighted median	0.16	0.08	.041	1.17 (1.01–1.37)	
*E. fissicatena* group	9	Simple mode	0.18	0.13	.200	1.20 (0.93–1.55)	
*E. fissicatena* group	9	Weighted mode	0.18	0.13	.195	1.20 (0.93–1.54)	
Ruminococcaceae UCG004	11	IVW	−0.16	0.08	.040	0.85 (0.72–0.99)	21.56
Ruminococcaceae UCG004	11	MR-Egger	−0.88	0.45	.079	0.41 (0.17–0.99)	
Ruminococcaceae UCG004	11	Weighted median	−0.13	0.11	.226	0.88 (0.71–1.08)	
Ruminococcaceae UCG004	11	Simple mode	−0.08	0.17	.628	0.92 (0.66–1.28)	
Ruminococcaceae UCG004	11	Weighted mode	−0.09	0.17	.606	0.91 (0.65–1.28)	

CI = confidence interval, IVW = inverse-variance weighted, MR = Mendelian randomization, OR = odds ratio, SE = standard error, SNP = single-nucleotide polymorphism.

### 3.3. Sensitivity analysis

**Figure 2.**
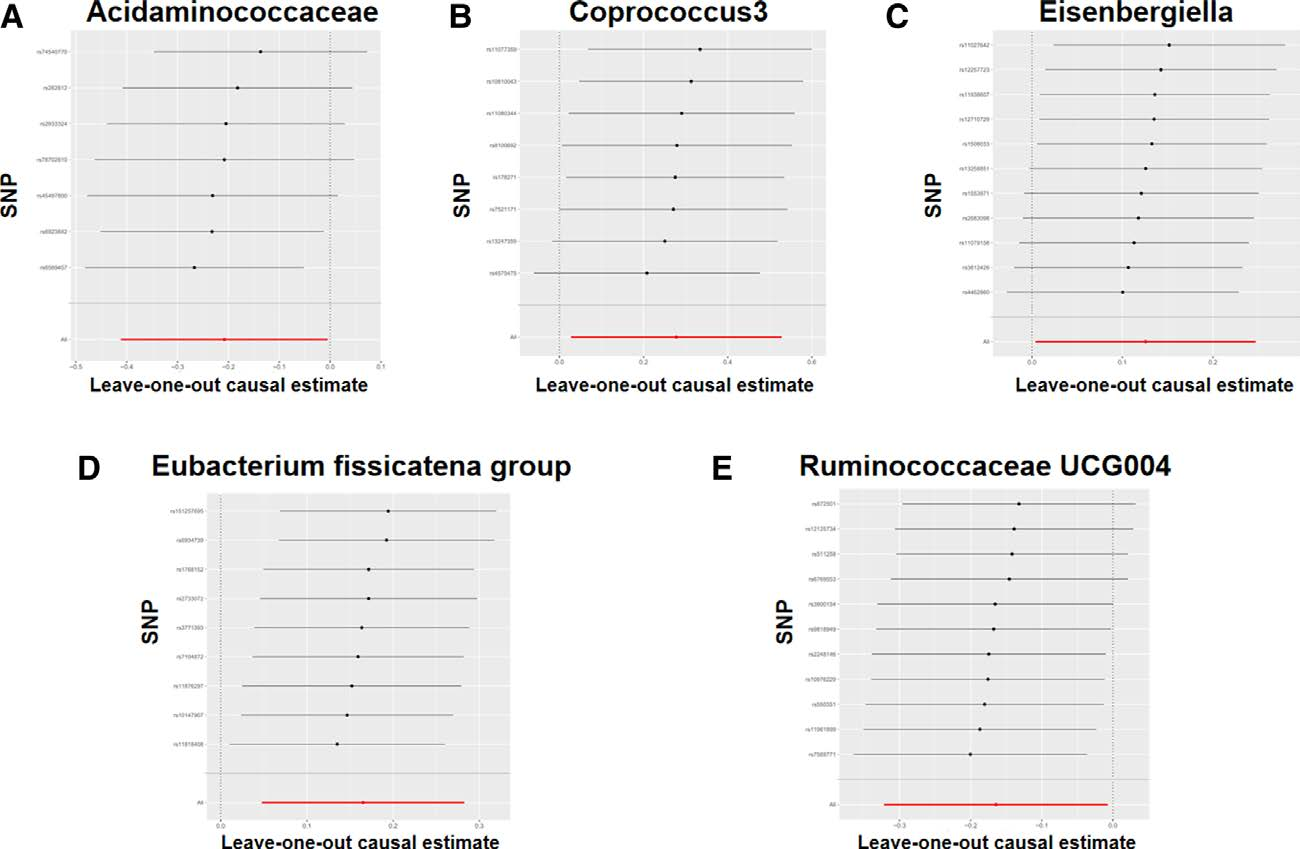
“Leave-one-out” analysis. The red lines are the analysis results of random-effects IVW. (A) Acidaminococcaceae and acute pancreatitis; (B) Coprococcus 3 and acute pancreatitis; (C) Eisenbergiella and acute pancreatitis; (D) *Eubacterium fissicatena* group and acute pancreatitis; (F) Ruminococcaceae UCG004 and acute pancreatitis. IVW = inverse-variance weighting, SNP = single-nucleotide polymorphism.

**Figure 3.**
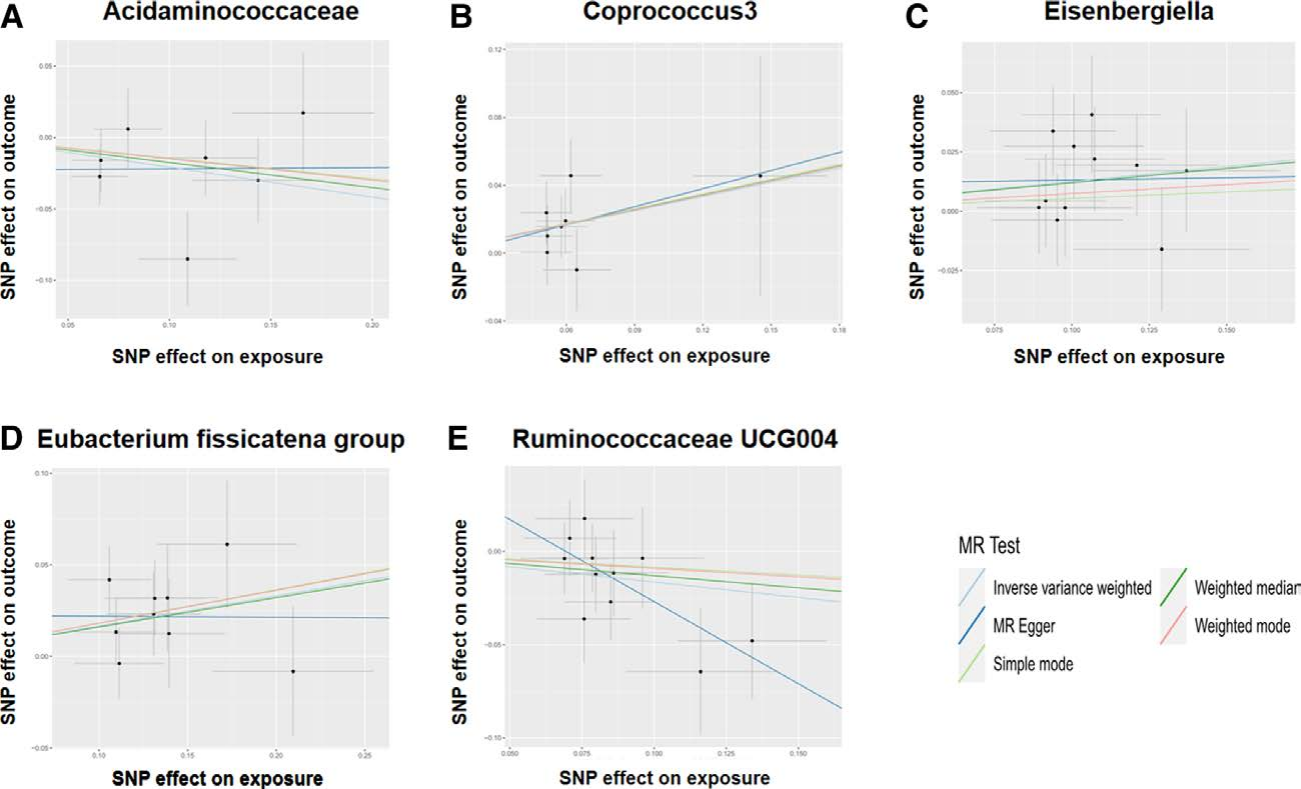
Scatter plots of the MR analyses for the association of 5 gut microbiota taxa and the risk of AP. (A) Causal effect of Acidaminococcaceae on AP; (B) causal effect of Coprococcus 3 on AP; (C) causal effect of Eisenbergiella on AP; (D) causal effect of the *Eubacterium fissicatena* group on AP; (F) causal effect of Ruminococcaceae UCG004 on AP. AP = acute pancreatitis, MR = Mendelian randomization, SNP = single-nucleotide polymorphism.

To improve the reliability of the MR analysis results, we performed a sensitivity analysis. First, MR-Egger regression-regression intercept analyses revealed no level of pleiotropy (*P* > .05) for the selected IVs. Similar results were obtained for the MR-PRESSO global test (Supplementary Table S2, Supplemental Digital Content, http://links.lww.com/MD/M669). Second, no heterogeneity was detected between the selected IVs according to Cochran test (*P* > .05, Table 2). Furthermore, the results of the retention method additionally supported the reliability of the sensitivity analysis (Figs. 2 and 3).

**Table 2:** Sensitivity analysis between gut microbiome and acute pancreatitis.

Exposure	Heterogeneity				Horizontal pleiotropy		
	Rucker *Q* MR-Egger	*P* value	Cochran *Q* IVW	*P* value	Egger-intercept MR-Egger	SE	*P* value
Acidaminococcaceae	5.793	.327	6.361	.384	−0.023	0.033	.515
Coprococcus 3	3.876	.693	3.888	.793	−0.005	0.041	.917
Eisenbergiella	5.963	.744	6.017	.814	0.011	0.048	.821
*E. fissicatena* group	5.603	.587	5.918	.656	0.023	0.040	.593
Ruminococcaceae UCG004	4.770	.854	7.444	.683	0.061	0.037	.1364

IVW = inverse-variance weighted, MR = Mendelian randomization, SE = standard error.

### 3.4. Reverse MR analysis

To prevent reverse causality from interfering with these results, we performed reverse MR analyses between AP and 5 gut microbial taxa by IVW as follows: Acidaminococcaceae (OR: 1.03, 95% CI: 0.88–1.20, *P* = .727), Ruminococcaceae UCG004 (OR: 1.07, 95% CI: 0.94–1.22, *P* = .311), Coprococcus 3 (OR: 1.04, 95% CI: 0.94–1.15, *P* = .470), Eisenbergiella (OR: 1.00, 95% CI: 0.83–1.21, *P* = 981), and the *E. fissicatena* group (OR: 1.07, 95% CI: 0.87–1.32, *P* = .508). Ultimately, we did not find a causal relationship between AP and the gut bacterial flora described above, as detailed in Supplementary Table S3, Supplemental Digital Content, http://links.lww.com/MD/M670.

## 4. Discussion

In recent years, a growing body of research has revealed a strong association between the gut microbiota and pancreatitis.^[22,23]^ For example, Chen et al^[24]^ reported that the diversity of the gut microbiota in acute necrotizing pancreatitis rats was significantly lower than that in controls and that the abundance of Tenericutes and Saccharibacteria was significantly increased in an animal model of AP. Tan et al^[25]^ reported a positive correlation between serum levels of IL-6 and serum levels of endotoxins and enterococcal abundance, suggesting that the inflammatory response may be associated with an imbalance in the gut microbiota. Lei et al^[26]^ have shown that acetate-producing Bacteroides alleviate heparenzymal exacerbations of AP by decreasing neutrophil infiltration. Li et al^[27]^ reported that Saikosaponin A reduces the severity of AP by increasing the relative abundance of *Lactobacillus* and *Prevotella* species.

However, there are many confounding factors in observational studies, resulting in an unclear causal relationship. This study explored the potential causal relationship between gut microbes and AP from a genetic perspective.

In this study, we performed MR analysis using the GM and AP GWAS databases to examine the causal relationship between the two. Our study showed that Acidaminococcaceae and Ruminococcaceae UCG004 were considered protective, whereas Eisenbergiella, Coprococcus 3, and the *E. fissicatena* group were associated with an increased risk of AP. The 5 gut taxa identified in this study are rarely reported to be involved in the development of AP. However, it has been reported that the gut microbiota plays an important role in some inflammatory diseases.

The *E. fissicatena* group is a gut pathogen.^[28]^ Liu et al pointed out that Bitter Panax ginseng flowers can target regulatory NF-KB signaling to suppress pathogenic bacteria, including the *E. fissicatena* group, thereby reducing colitis.^[29]^ Another study by Li et al^[28]^ also revealed that *Rabdosia Serra* improved colitis progression in mice by reducing the abundance of the *E. fissicatena* group. Ruminococcaceae UCG004 has thick-walled hyphae and can repair the intestinal barrier and inhibit inflammatory progression by producing short-chain fatty acids (SCFAs).^[30,31]^ Based on the systemic nature of the lung-gut axis, Mazumder et al^[32]^ reported that lung inflammation increased the burden on the gut, resulting in significant changes in the mRNA expression of Ruminococcaceae and its metabolites in C57BL/6J mice exposed to air pollution.

Trimethylamine n-oxide (TMAO) is an important gut microbiota-derived compound,^[33]^ and elevated TMAO is strongly associated with exacerbated inflammation and oxidative stress.^[34,35]^ Huo et al^[36]^ recently reported a significant positive correlation between Coprococcus 3 and increased TMAO in the urine of 52 patients with T2DM and 15 healthy controls. In addition, Liu et al^[37]^ found that Coprococcus 3 is closely associated with gallstone disease and gallstone cholecystitis from a genetic perspective. Interestingly, cholelithiasis is a major cause of AP.^[38]^ However, Vijay et al^[39]^ showed that Coprococcus 3 inhibits the progression of inflammation by producing SCFAs. These conflicting results may be attributed to the limitations and confounders of this observational study. Our genetic analysis suggested that Coprococcus 3 is a risk factor for AP and may exacerbate the predisposition to AP. The underlying mechanism remains to be determined.

Acidaminococcaceae can produce SCFAs.^[40]^ To date, the biological effect of Acidaminococcaceae on AP has not been investigated. However, it has been shown that the gut microbiota can promote the expression of IL-10, which has anti-inflammatory effects, in macrophages by producing SCFAs, thereby reducing the progression of inflammation-related diseases.^[41,42]^ In addition, SCFAs enhance the anti-inflammatory properties of epithelial cells by inhibiting the activity of histone deacetylase, which is a ligand for the GPR41 and GPR43 receptors.^[43,44]^ These studies suggest that Acidaminococcaceae can inhibit the inflammatory process by producing SCFAs. Interestingly, Acidaminococcaceae has also been shown to regulate lipids by reducing the accumulation of lipids in the cardiovascular system and changing immune inflammation.^[45]^ Notably, hyperlipidemia is an important cause of AP.^[46,47]^

Eisenbergiella bacteria exhibit pathogenic properties in some inflammatory diseases.^[48,49]^ However, its role in AP has not been studied. In this study, we found that the presence of Eisenbergiella is a pathogenic factor for AP. In a study that evaluated 98 patients with gestational diabetes mellitus and 98 healthy controls, a randomized controlled trial revealed high expression of Eisenbergiella in the gut microbiota of GDM subjects, and it was positively correlated with fasting blood glucose levels. Interestingly, there is a strong association between diabetes and pancreatitis.^[50,51]^ Overconsumption of fatty acids in healthy people can lead to a shift to diseases such as inflammation and cancer.^[52,53]^ Bailén et al^[54]^ reported significantly greater expression of Eisenbergiella in men fed a high-fat diet, suggesting that it may be involved in lipid regulation. In addition, an animal study revealed that in mice, tributyltin (TBT)-mediated inflammation of the liver was further exacerbated by the presence of Eisenbergiella through the regulation of abnormalities in TGs and other lipids.^[55]^ Based on these studies, we speculate that Eisenbergiella may contribute to the development of pancreatitis by regulating lipid abnormalities. The specific mechanism needs to be further studied.

Notably, the reliability of our results was further confirmed by a recent study by Wang et al^[56]^ The difference is that we used a larger and more comprehensive database to make our results more reliable. In addition, we found that 2 gut microbial taxa, Acidaminococcaceae and Eisenbergiella, had a significant causal relationship with AP. The protective mechanism of Acidaminococcaceae and the pathogenic mechanism of Eisenbergiella need to be further investigated and are expected to be new targets for the clinical treatment of AP.

The major advantage of this study is that we used MR analysis together with the largest public GWAS dataset, and the results of the MR analysis were less influenced by confounders and reverse causality. Furthermore, we conducted a randomized reverse Mendelian study and found no evidence of a reverse causal relationship. This makes our findings more convincing.

This study has several limitations. Although our study satisfies the MR hypothesis, it does not guarantee a weak tool bias. Although the GWAS dataset is large enough to include only subjects of European ancestry, it may not apply to subjects from other regions.

## 5. Conclusions

In conclusion, we identified a significant causal relationship between 5 gut microbial taxa and AP using 2-sample MR analysis, providing a new target for the clinical treatment of AP.

## Acknowledgments

The authors thank the participants of all GWAS cohorts included in the present work and the investigators of the MiBioGen and FinnGen initiatives for sharing the GWAS summary statistics.

## Author contributions

**Data curation:** Lin He.

**Methodology:** Lin He.

**Writing—original draft:** Lin He.

**Writing—review & editing:** Hang Liu.

**Funding acquisition:** Haojun Luo, Hang Liu.

**Project administration:** Hang Liu.

**Investigation:** Yu Li.

**Resources:** Haojun Luo.

**Visualization:** Lin He,Yan Xu.

**Validation:** Yan Zhang, Li Peng, Yan Xu, Jing Lu, Jinzhi Li.

## Supplementary Material






